# Data on THMs concentration and spatial trend in water distribution network (a preliminary study in center of Iran)

**DOI:** 10.1016/j.mex.2019.03.030

**Published:** 2019-04-03

**Authors:** Amir Mohammadi, Ali Asghar Ebrahimi, Reza Ghanbari, Maryam Faraji, Sepideh Nemati, Ali Abdolahnejad

**Affiliations:** a Department of Public Health, Maragheh University of Medical Sciences, Maragheh, Iran; b Student Research Committee, Shahid Sadoughi University of Medical Sciences, Yazd, Iran; c Environmental Science and Technology Research Center, Department of Environmental Health Engineering, Shahid Sadoughi University of Medical Sciences, Yazd, Iran; d Social Determinants of Health Research Center, Qazvin University of Medical Sciences, Qazvin, Iran; e Environmental Health Engineering Research Center, Kerman University of Medical Sciences, Kerman, Iran; f Department of Environmental Health, School of Public Health, Kerman University of Medical Sciences, Kerman, Iran; g Health Faculty, Student Research Committee, Tabriz University of Medical Sciences, Tabriz, Iran

**Keywords:** USEPA Method 524.2 (1995) by GC/MS, Kriging mapping method, Water, THMs, Monitoring, Spatial trend

## Abstract

The Trihalomethanes (THMs) formed due to a reaction between water disinfection chlorine and some natural organic matters, as chlorinated by products. The aim of this study was determination of THMs values and spatial trend in Yazd city water distribution network, in center of Iran. Sampling of tap water was done in two autumn and winter seasons. The THMs value were measured by using a gas chromatograph-mass spectrometer (GC/MS), Agilent Company 6890 N. The spatial analysis of THMs values was carried out using ArcGIS 10.1 to show the spatial spreading. The Kriging method was used to draw distribution maps. Using the Kriging method to illustrate the difference or precision of forecasts is relatively easy compared to the other interpolation methods. Also, the acceptable level of % RMSE (Root mean square error) was calculated for Kriging method (% RMSE > 40). Thus, this protocol as integrated between data and geraphic could easily used for reporting of THMs level in studies of water distribution network.

Finally, the maximum THMs value were obtained lower than USEPA and WHO guidelines for drinking water (THMs < 40 ppb).

## Graphical abstract

**Figure:**
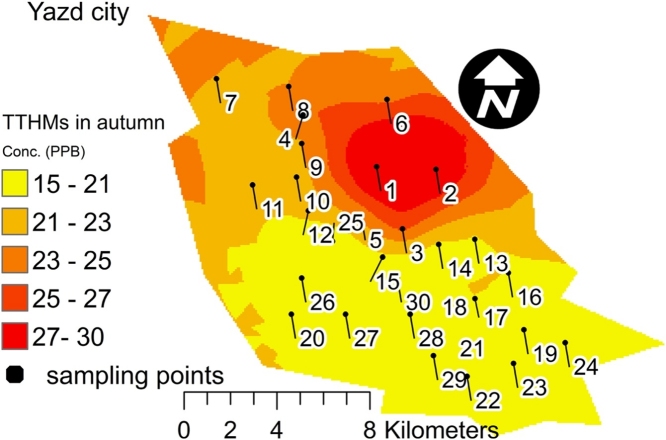

**Table:** 

**Subject Area**	*Environmental Science*
**Protocol name:**	*USEPA Method 524.2 (1995) by GC/MS, Kriging mapping method*
**Reagents/tools:**	*GC/MS, column characteristics: model: Agilent LS DB5-MS, type: capillary, length: 30 m, internal diameter: 0.25 mm; injector characteristics: injection volume: 1500 μL HS 10* ml *sample, temperature: 160 °C; mode: injector split, and carrier gas: helium 1 mL/minute*
*Experimental design:	*All sampling and parameters analysis were measured according to in Standard Methods for the Examination of Water and Wastewater.*
*Value of the Protocol:	*Trihalomethanes (THMs) produced in treated water due to the reaction between of chlorine and some natural organic matters. These byproducts compounds could lead to bladder, kidney, and liver cancers* [1,2]. *The obtained data about THMS with Arc GIS could be used for better understand the quality water distribution network in association with disinfection byproduct values in the study area.* *Based on the data, the water in the study area in period of the study was based on the WHO and USEPA guideline, thus it appropriate for drinking* [3,4].

## Description of protocol

### Study area and water characteristics

Yazd is one of the oldest cities in the center of Iran (31º 88ʹ N, 54º 360ʹ E). The area and population of this city are about 110 km^2^ and 656,000 person according to 2016 census [5]. For selection of sampling sites, city zoned in 30 sites. The sources of water distribution network in Yazd city include the surface water of Zayandehroud River and groundwater from wells.

### Sampling procedure and field measurements

Sampling procedure was carried out in the autumn and winter seasons 2017, 30 samples were taken from water distribution network during each season, according to standard methods for water and wastewater examination [6]. Prior to the sampling, bottles (300-mL amber glass) were washed using detergent–deionized water, and then dried in an oven at 400 °C. Furthermore, sodium thiosulfate was added to the sampling bottles for removal of disinfection chlorine and inhibition of THMs formation during samples transportation and storage. Sampling process was done in official and commercial buildings using tap water. Also pH, EC and residual chlorine (Table 1) measured in the sampling place by pH meter 310 AUTECH and chlorine meter model 5f8700-12 HACH [7,8].

**Table 1:** THMs concentration (μg/L) in water distribution network of Yazd city, center of Iran.

Samples (N: 30)	TTHMs	CHBr_3_		CHBr_2_Cl	CHBrCl_2_	CHCl_3_	Cl_2_ (ppm)	EC (μs/cm)	pH (±0.2)	TOC (ppm)
	(ppb)									
autumn season										
Mean	21.53	4.73		4.9	4.8	7.07	0.50	456.63	7.11	<1
Standard deviation	3.34	0.78		0.4	0.53	3.24	0.11	70.85	0.18	<1
Max	31.00	7.00		5.00	5.00	17.00	0.80	596.00	7.47	<1
Min	18.00	3.00		4.00	3.00	3.00	0.2	365.00	6.84	<1
Temperature	23 ± 2 °C									
Winter season										
Mean	22.61	4.86		4.9	5.13	8.80	0.72	447.93	7.90	<1
Standard deviation	5.12	0.5		0.48	0.35	4.62	0.14	73.52	0.20	<1
Max	39.00	5.00		6.00	6.00	23.00	1.10	588.00	8.30	<1
Min	18.00	3.00		3.00	5.00	5.00	0.14	329.00	7.60	<1
Temperature	14 ± 2 °C									
WHO guideline for THMs			([TTHMs/WHO guideline] ≤ 1)							
USEPA guideline for THMs			80 (μg/L)							

### Determination of THMs

THMs analyzed based on Method 524.2 (USEPA 1995) by a gas chromatograph-mass spectrometer (GC/MS), Agilent Company 6890 N, and a purge and trap system [9]. This method is approvable for USEPA and can be used in the countries.

In the first step, standard samples of THMs compounds were made in concentrations of 1–100 ppb by diluting THMs standard solution (200 μg/ml), which was purchased from Sigma Aldrich company. Then, peak curves of four THMs compounds were taken from GC/MS analysis, and the calibration curve was drawn using Microsoft Excel program.

### In the second step, water network samples were analyzed using GC/MS

Some information about the GC/MS analysis include Column characteristics: model column characteristics DB5-MS, type capillary, length 30 m, internal diameter 0.25 mm, Injector characteristics: Injection volume 1500 μl HS 10 cc sample, temperature 160 °C, mode injector split, carrier gas Heluim 1 ml/min. More details is available in our previous study [10]. Also, samples and blanks were measured in duplicates for quality assurance. The relative standard deviation (RSD) values lower than 30% were accepted. Recoveries of standard reference compound and external stands changed from 91.6%–95.2%, and the detection limit was 0.5 μg/L.

### Spatial analysis method

**Fig. 1:**
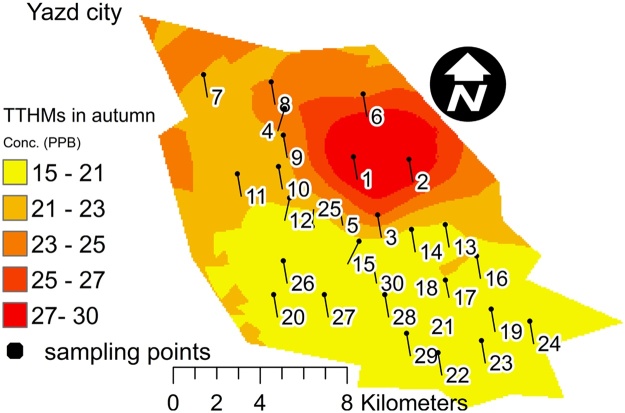
Spatial mapping of THMs value in autumn.

**Fig. 2:**
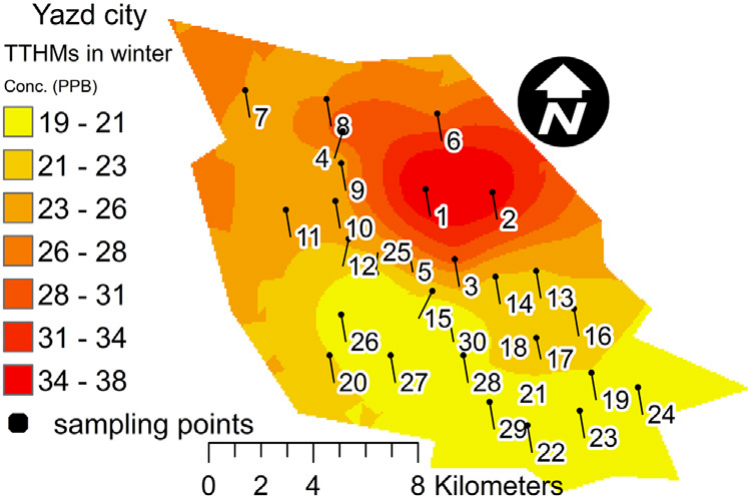
Spatial mapping of THMs value in winter.

To more assess the values of THMs in the water distribution network, THMs distribution maps were created in ArcGIS to obtain the spatial spreading. The Kriging interpolation method was used to draw independent raster layers of THMs values. Also, the raster calculator function was used to overlay each layer to produce maps for two seasons. To choose the best prediction approach, the acceptable value of % RMSE was evaluated for Kriging method (% RMSE > 40). The areas showing high and low levels of THMs pollution were highlighted by several dimension stretch style (Fig. 1, Fig. 2).

The main advantages of THMs spatial mapping are classified color and easy optical detection. The THMs concentrations in the red area were higher than those in the yellow area. Thus, this protocol could be easily used for reporting of quality and quantity THMs in water distribution network. Furthermore, the application of the data and graphical report can be very interesting.
